# Phosphoinositide specific phospholipase Cγ1 inhibition-driven autophagy caused cell death in human lung adenocarcinoma A549 cells *in vivo* and *in vitro*

**DOI:** 10.7150/ijbs.42962

**Published:** 2020-02-21

**Authors:** Xiaohong Lu, Haijing Fu, Rui Chen, Yue Wang, Yanyan Zhan, Gang Song, Tianhui Hu, Chun Xia, Xuemei Tian, Bing Zhang

**Affiliations:** 1 Cancer Research Center, School of Medicine, Xiamen University, 361102, Fujian, China; 2 Zhongshan Hospital, Xiamen University,361004, Xiamen, Fujian, China; 3 School of Life Sciences, South China Normal University, 510631, Guangzhou, Gangdong, China

**Keywords:** PLCγ1 inhibition, autophagic cell death (ACD), lung adenocarcinoma A549 cells, A549 xenograft nude mice

## Abstract

Our previous studies indicated that phosphoinositide specific phospholipase Cγ1 (PLCγ1) was involved in autophagy induction in colon and hepatic carcinoma cells. However, whether and how PLCγ1 regulation in human lung adenocarcinoma is linked to autophagy remains unclear. Here, we assessed the protein expression of PLCγ1 in human lung adenocarcinoma tissue using immunohistochemistry assay and the relationship between PLCG1 and autophagy in The Cancer Genome Atlas Network (TCGA) using Spearman correlation analysis and GSEA software. Furthermore, the interaction between PLCγ1 and autophagy-related signal molecules was investigated in human lung adenocarcinoma A549 cells treated with different inhibitors or transduction with lentivirus-mediated PLCγ1 gene short-hairpin RNA (shRNA) vectors using MTT, clonogenicity, Transwell migration, RT-PCR, Caspase-3, mitochondrial transmembrane potential, and western blotting assays, as well as transmission electron microscope technique. Additionally, the effect of shRNA/PLCγ1 alone or combined with autophagic activator Lithium Chloride (LiCl) on tumor growth and metastasis was measured using immunohistochemistry and assays in A549 xenograft nude mouse model. The results showed that increased PLCγ1 expression occurred frequently in human lung adenocarcinoma tissue with higher grades of T in TNM staging classification. PLCγ1 significantly enriched in autophagic process and regulation, which negatively regulating autophagy was enriched in higher expression of PLCγ1. PLCγ1 inhibition partially reduced cell proliferation and migration of A549 cells, with an increased autophagic flux involving alterations of AMPKα, mTOR, and ERK levels. However, PLCγ1 inhibition-driven autophagy led to cell death without depending on Caspase-3 and RIP1. Additionally, the abrogation of PLCγ1 signaling by shRNA and combination with autophagic activator LiCl could efficaciously suppress tumor growth and metastasis in A549 xenograft nude mice, in combination with a decrease in P62 level. These findings collectively suggest that reduction of cell proliferation and migration by PLCγ1 inhibition could be partially attributed to PLCγ1 inhibition-driven autophagic cell death (ACD). It highlights the potential role of a combination between targeting PLCγ1 and autophagy pathway in anti-tumor therapy, which may be an efficacious new strategy to overcome the autophagy addition of tumor and acquired resistance to current therapy.

## Introduction

Macroautophagy (afterwards referred to as autophagy) could serve as a cytoprotective mechanism in response to various pathological status and stress, including tumor 1-4. At the early stage of tumor, autophagy links to suppression of tumor development; autophagy actuates progression and therapy resistance by maintaining cellular homeostasis in fully evolved tumors 2, 3. Hence, autophagy is a double-edged sword in tumor process 4. Although targeting autophagy could be a promising therapeutic approach for tumor treatment through blocking development, preventing therapy resistance, and improving clinical outcome 5, treating patients with autophagy inhibitors or enhancers limits on experimental stage with an unfavorable outcome [3,6.7]. It may be associated with the ambiguous biological mechanism of how autophagy affects tumor initiation, progression, and therapy 6.

Phosphoinositide specific phospholipase Cγ1 (PLCγ1) plays an important role in regulating cell proliferation and migration of tumor 8-10. Very recent study reports that PLCG1 could act as an oncogene in hepatocellular carcinoma carcinogenesis and may serve as a valuable prognostic marker and potential therapeutic target for hepatocellular carcinoma 11. Some studies have addressed the existence of a link between PLCγ and autophagy. Both IP3 and DAG (two hydrolysis products of PIP2 by PLCγ activity) are involved in regulating autophagy 12-14. Additionally, in primary human keratinocytes, PLCγ is the FGFR2b substrate, which acts as an upstream regulator of both phagocytosis and autophagy 15. Our previous study also demonstrated that PLCγ1 inhibition could induce autophagy in HCT116 and HepG2 cells 16. Hence, there exists a link between PLCγ1 and autophagy in tumor progression.

Lung adenocarcinoma is 40% of total lung cancer cases, which has a 5-year survival rate of only 15% 17. PLCγ1 as one of oncogenic HER2 downstream signaling mediators is hyper-activated in HER2H878Y driven lung tumors 18; PLCγ is associated with bulk migration of non-small cell lung carcinomas 19. Recently, Zahra Timsah et al reported that expression pattern of PLCγ1 acts as a novel prognostic marker of recurrence-free survival in lung adenocarcinoma 20. Therefore, it is conceivable that PLCγ1 participates in regulating cell metabolism of lung carcinoma. However, whether and how PLCγ1 regulation is linked to autophagy has not been determined in human lung adenocarcinoma.

In this study, we investigated the effect of PLCγ1 inhibition on cell proliferation and migration and its regulatory mechanism linked to autophagy in human lung adenocarcinoma A549 cells. Furthermore, the effect of shRNA/PLCγ1 alone and combination with autophagy activator Lithium Chloride (LiCl) on tumor growth and metastasis was evaluated in A549 xenograft nude mouse model. Our studies demonstrated that PLCγ1 inhibition-driven autophagy led to autophagic cell death (ACD), which might partially take responsibility for the reduction of cell proliferation and migration by PLCγ1 inhibition. This highlights a potential role of the combination of targeting PLCγ1 and autophagy pathway in antitumor therapy.

## Results

### PLCγ1 inhibition reduced cell proliferation and migration in human adenocarcinoma A549 cells

**Figure 1:**
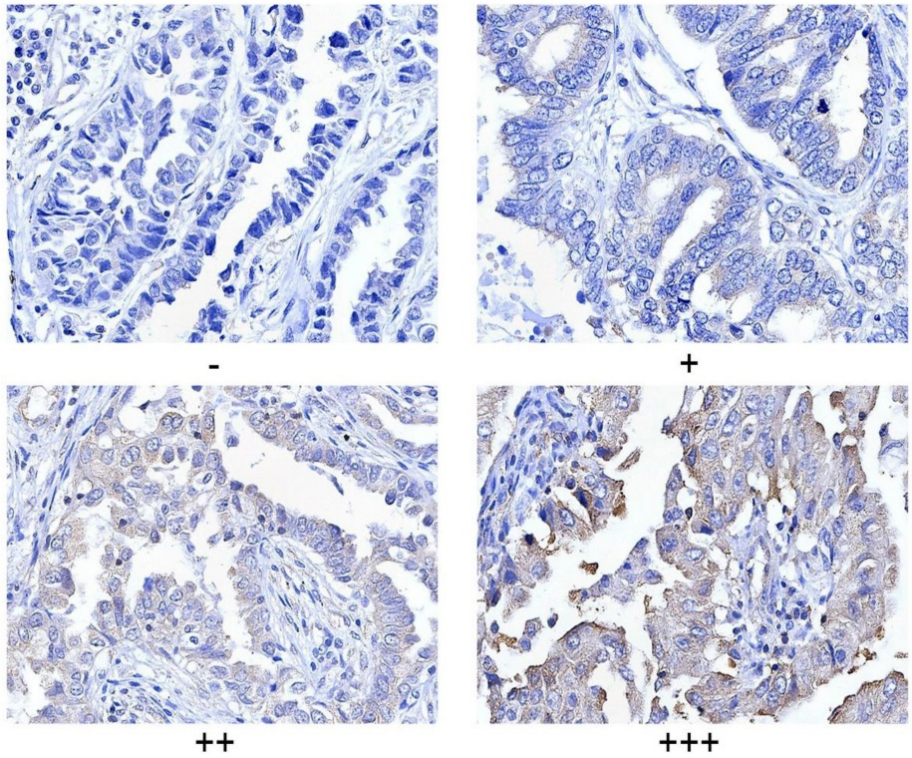
** Criteria for statistical analysis of PLCγ1 expression in human adenocarcinoma.** PLCγ1 expression was detected in the tissue microassay with immunohistochemical assay as described in the Material and methods section (original magnification × 40).

**Figure 2:**
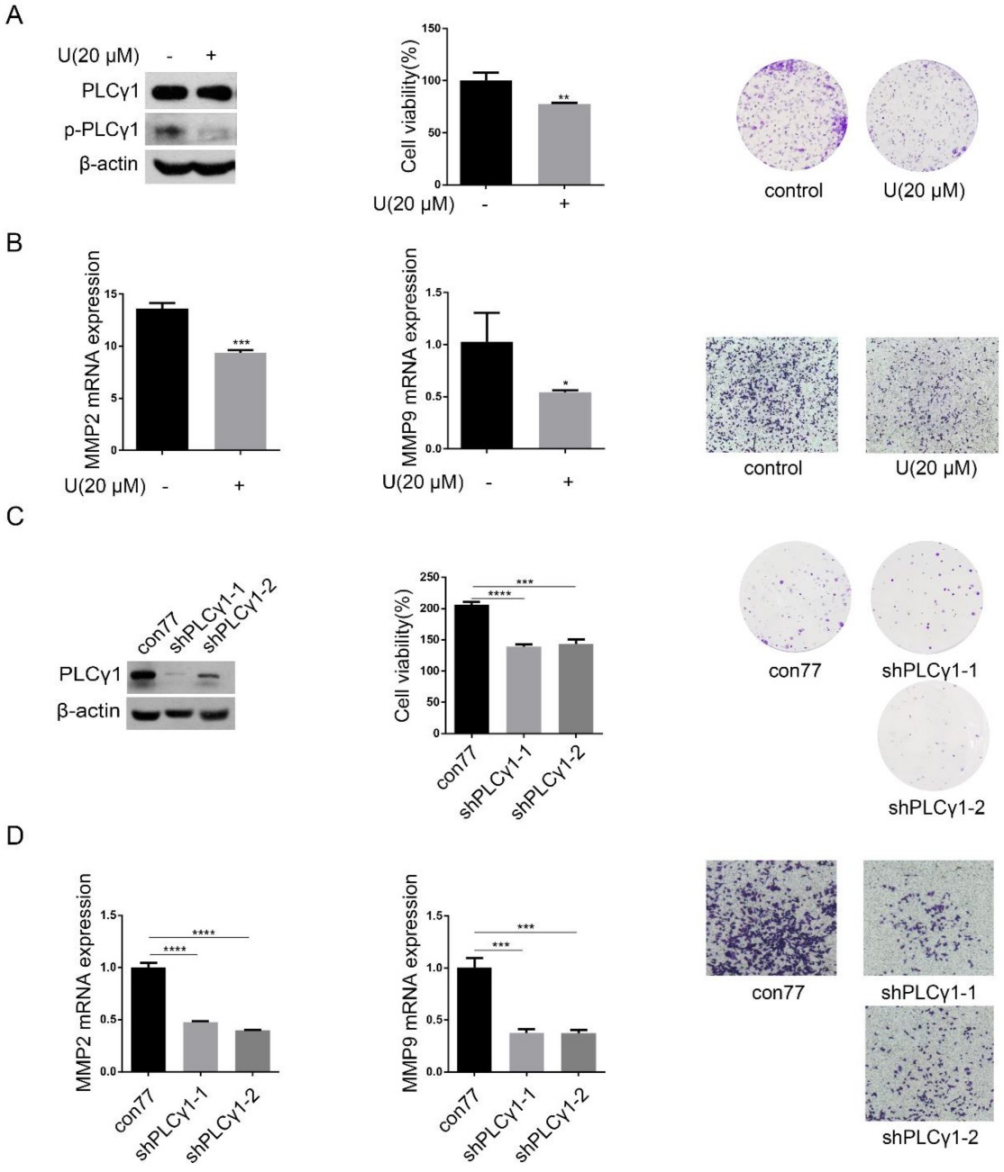
** Effect of PLCγ1 on cell proliferation and migration of human adenocarcinoma A549 cells.** (A) & (B) Cells were treated with U (20 μM) for 24 hours. The PLCγ1, p-PLCγ1, and β-actin protein levels were detected via western blotting, followed with the detection of cell viability via MTT assay and cell growth via colony forming as described in the Material and methods section(A). The MMP2 and MMP9 mRNA levels were detected via RT-PCR assay and the migrated cells were observed via Transwell migration assay as described in the Material and methods section (B). (C)&(D) Cells were transduced with shRNA/PLCγ1-1/2 vectors. The PLCγ1 and β-actin protein levels were detected via western blotting, followed with the detection of cell viability via MTT assay and cell growth via colony forming as described in the Material and methods section(C). The MMP2, MMP9, and GAPDH mRNA levels were detected via RT-PCR assay and the migrated cells were observed via Transwell migration assay as described in the Material and methods section (D). The data are representative of three independent experiments (*p<0.05, **p<0.01, ***p<0.001, ****p<0.0001).

PLCγ1 expression in human lung adenocarcinoma was assessed by immunohistochemistry assay in a tissue microarray. The majority (78.57%) of human lung adenocarcinoma specimens (T2, T3) showed a specific immune reactivity to PLCγ1 (Fig.1, Table 1, p=0.003<0.05). Although the percentage of higher expression of PLCγ1 in human lung adenocarcinoma specimens with regional lymph node metastasis (N1, N2, 73.91%) was more than that in non-metastatic specimens (N0,48.48%), there was no significance between them, maybe due to the smaller number of samples in the tissue microarray (Table 1, p=0.057>0.05). As a result, increased PLCγ1 expression occurred frequently in human lung adenocarcinoma tissue with higher grades of T in TNM staging classification. Meanwhile, human lung adenocarcinoma A549 cells were treated with PLCγ1 inhibitor U73122 (U), following the detection of cell viability and migration. Treatment of U significantly reduced cell viability and colony formation (Fig. 2A, **p<0.01, *vs* untreated group). The mRNA level of MMP2 and MMP9 and number of migrate cells decreased significantly in A549 cells in response to U (Fig.2B, *p<0.05, ***p<0.001,* vs* untreated group). Similarly, the depletion of PLCγ1 with lentiviral-mediated shRNA/PLCγ1-1/2 (shPLCγ1-1/2) vectors caused a reduction of cell proliferation and migration (Fig.2C&D, ***p<0.001, ****p<0.0001,* vs* con77 group). Taken together, the data indicated that increased PLCγ1 expression occurred frequently in human lung adenocarcinoma tissue with higher grades of T in TNM staging classification and that PLCγ1 inhibition reduced cell proliferation and migration in human lung adenocarcinoma A549 cells.

### PLCγ1 inhibition promoted autophagic flux in human lung adenocarcinoma A549 cells

**Figure 3:**
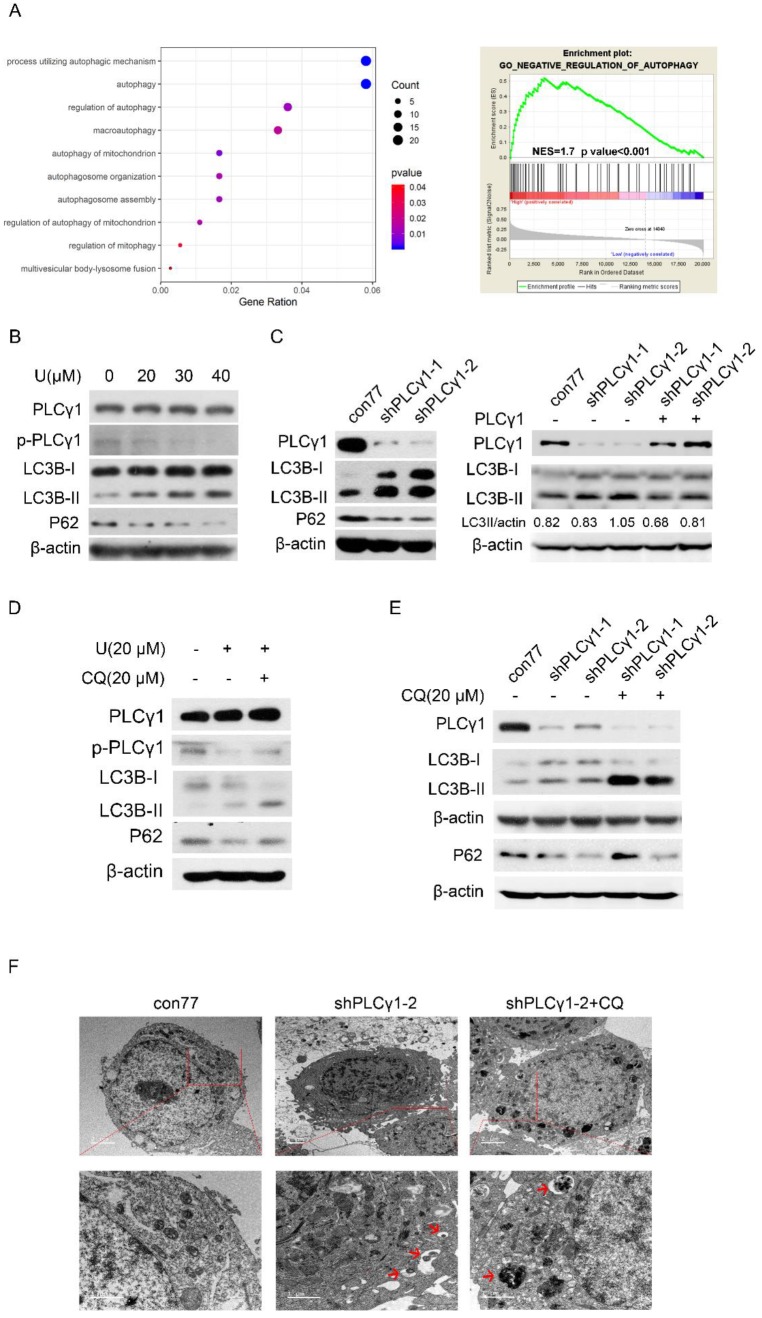
** PLCγ1 inhibition promotes autophagic flux in human adenocarcinoma A549 cells.** (A) The relationship between PLCG1 and autophagy was assessed in The Cancer Genome Atlas Network (TCGA) by Spearman correlation analysis and GSEA software as described in the Material and methods section (r≤-0.4, p<0.05). (B) Cells were treated with different concentrations of U (20, 30, and 40 μM) for 24 hours and the PLCγ1, p-PLCγ1, LC3B, p62, and β-actin protein levels were detected via western blotting as described in the Material and methods section. (C) Cells were transduced with shRNA/PLCγ1-1/2 vectors and the PLCγ1, LC3B, p62, and β-actin protein levels were detected via western blotting as described in the Material and methods section (Left panel). Cells stably expressing shRNA/PLCγ1-1/2 were transiently transfected with pRK5-PLCγ1 vectors, and the PLCγ1, LC3B, and β-actin protein levels were detected via western blotting (Right panel). (D) Cells pretreated with CQ (20 μM) for 1 hour were co-treated with U (20 μM) for 24 hours and the PLCγ1, p- PLCγ1, LC3B, p62, and β-actin protein levels were detected via western blotting analysis as described in the Material and methods section. (E)&(F) Cells transduced with shRNA/PLCγ1-1/2 vectors were treated with or without CQ (20 μM) for 24 hours. The PLCγ1, LC3B, p62, and β-actin protein levels were detected via western blotting as described in the Material and methods section (E). After samples were made as described in the Material and methods section and autophagic vacuoles (indicated by red arrows) were observed under a transmission electron microscope (F). The data are representative of three independent experiments.

The relationship between PLCγ1 and autophagy was firstly assessed in The Cancer Genome Atlas Network (TCGA) by Spearman correlation analysis and GSEA software. Integrative and comparative genomic analysis of human lung adenocarcinoma demonstrated that PLCγ1 significantly enriched in autophagic process and regulation, which negatively regulating autophagy was enriched in higher expression of PLCγ1 (Fig. 3A, r≤-0.4, p<0.001). Furthermore, the expression level of autophagic markers, including LC3B and P62, was measured via western blotting analysis in A549 cells in response to U. Fig.3B showed that treatment of U led to an increase in LC3B-II level in combination with a decrease in P62 level, implying that U increased autophagic flux. Similar results were observed in A549 cells transduced with shPLCγ1-1/2 vectors (Fig. 3C, Left panel). The increased LC3B-II level by shPLCγ1-1/2 vectors was reversed by transfection with pRK5-PLCγ1 vectors (Fig. 3C, Right panel). Especially, addition of Chloroquine (CQ, autophagy inhibitor) interrupted the execution of PLCγ1 inhibitor on LC3B-II and P62 protein expression (Fig.3D&E). In addition, under a transmission electron microscope, autophagic vacuoles (red arrows) increased obviously without chromatin condensation and oncosis in A549 cells transduced with shPLCγ1-2 vector (Fig.3F). Overall, PLCγ1 inhibition using either shRNA vector or inhibitor promoted autophagic flux in A549 cells.

### Involvement of AMPKα, mTOR, and ERK signal molecules in PLCγ1 inhibition-driven autophagy in human lung adenocarcinoma A549 cells

**Figure 4:**
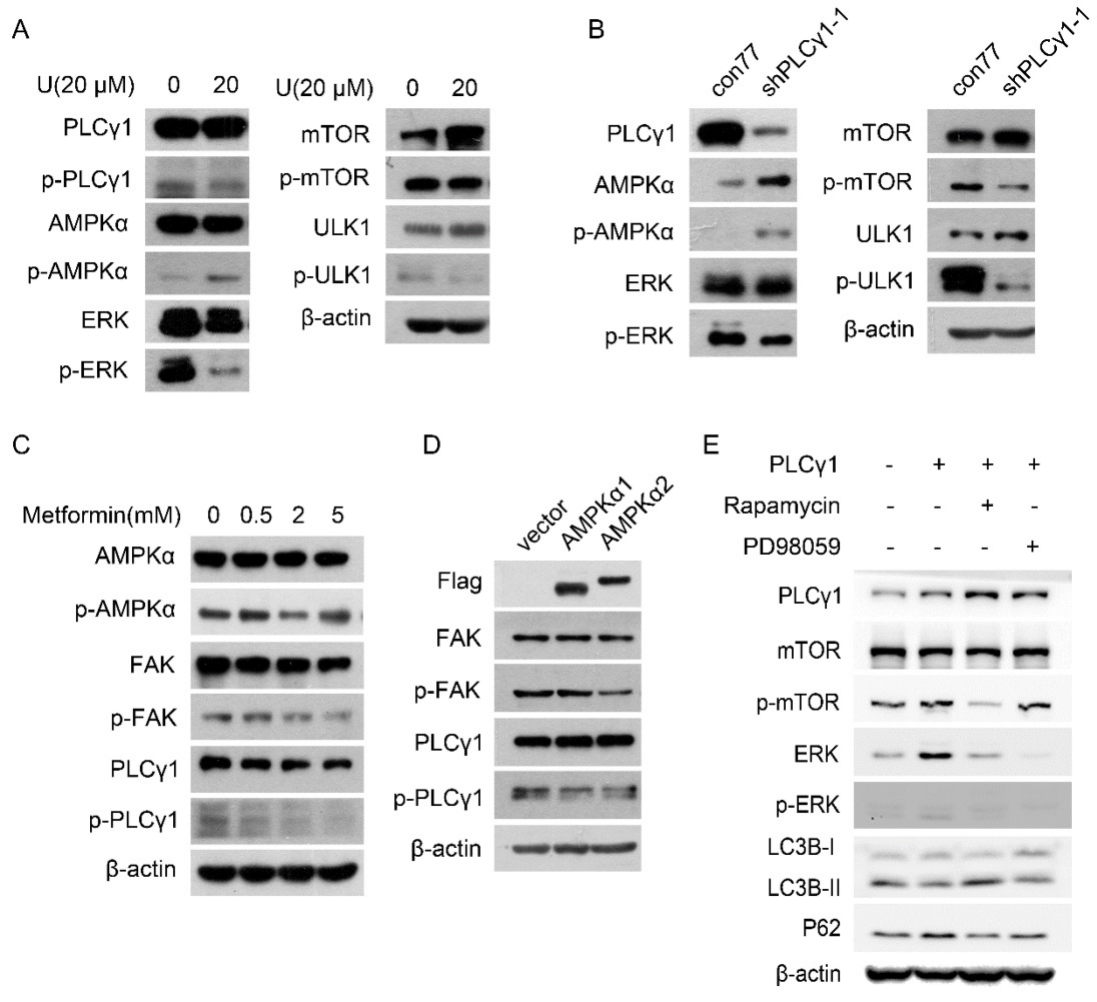
** Involvement of AMPK, mTOR, and ERK in PLCγ1 inhibition-driven autophagy in A549 cells.** (A) Cells were treated with U (20 μM) for 24 hours, and the PLCγ1, p-PLCγ1, AMPKα, p-AMPKα, mTOR, p-mTOR, ULK1, p-ULK1, ERK, p-ERK, and β-actin protein levels were detected via western blotting as described in the Material and methods section. (B) Cells were transduced with shRNA/PLCγ1-1 vector, and the PLCγ1, p-PLCγ1, AMPKα, p-AMPKα, mTOR, p-mTOR, ULK1, p-ULK1, ERK, p-ERK, and β-actin protein levels were detected via western blotting as described in the Material and methods section. (C) Cells were treated with different concentrations of Metformin (0.5,2, 5 mM) for 12 hours and the PLCγ1, p-PLCγ1, AMPKα, p-AMPKα, FAK, p-FAK, and β-actin protein levels were detected via western blotting as described in the Material and methods section. (D) Cells were transiently transfected with AMPKα1 or AMPKα2 vector, and the Flag, PLCγ1, p-PLCγ1, FAK, p-FAK, and β-actin protein levels were detected via western blotting as described in the Material and methods section.(E) Cells transfected transiently with pRK5-PLCγ1 vectors were treated with or without Rapamycin(1 μM) and PD98059 (20 μM) for 24 hours, respectively. The PLCγ1, mTOR, p-mTOR, ERK, p-ERK, LC3B, P62, and β-actin protein levels were detected via western blotting as described in the Material and methods section. The data are representative of three independent experiments.

Fig. 4A displayed that treatment with U elevated the phosphorylation of AMPKα at Thr172 site (p-AMPKα) in A549 cells. Transduction with shPLCγ1-1 vector has the same effect on p-AMPKα (Fig. 4B). In contrast, the phosphorylation of mTOR at Ser2448 site (p-mTOR), ULK1 at Ser757 site (p-ULK1), and ERK at Thr202/Tyr204 site (p-ERK) was interrupted by treatment with U or transduction with shPLCγ1-1 vector (Fig.4A, 4B).

To further confirm the relationship between AMPKα, mTOR, and ERK in PLCγ1 inhibition-driven autophagy, A549 cells were treated with different activator and inhibitor, or transfected with different expressing vectors. Fig. 4C showed that both p-PLCγ1 and p-FAK levels significantly decreased in A549 cells in response to 5 mM metformin (an AMPKα activator). Similarly, transfection with p3xFLAG-CMV10-AMPKα1 (AMPKα1) vector expressing AMPKα1 and p3xFLAG-CMV10-AMPKα2 (AMPKα2) vector expressing AMPKα2 caused an observable decrease in p-PLCγ1 and p-FAK levels (Fig.4D). Thus, AMPK might act upstream of PLCγ1 and FAK to execute its function in A549 cells. Meanwhile, A549 cells transfected with pRK5-HA-tagged PLCγ1 vector were treated with respective inhibitors of mTOR (Rapamycin) or ERK (PD98059). Fig. 4E showed that overexpressed PLCγ1 elevated p-mTOR and p-ERK levels, along with a decrease in LC3B-II and an increase in P62 levels, compared with un-transfected group with pRK5-HA-tagged PLCγ1 vector. Compared with A549 cells transfected with pRK5-HA-tagged PLCγ1 vector without Rapamycin or PD98059 treatment, treatment of Rapamycin partially reversed the effect of overexpressed PLCγ1 on LC3B-II and P62 levels, such as an increase in LC3B-II and a decrease in P62 levels (Fig.4E). Treatment of PD98059 had little influence on LC3B-II and P62 levels (Fig.4E). Additionally, Rapamycin not only suppressed p-mTOR, but also reduced ERK and p-ERK levels in A549 cells overexpressing PLCγ1. However, PD98059 had no obvious influence on p-mTOR level in A549 cells overexpressing PLCγ1 (Fig.4E). mTOR might be reasonably supposed to act upstream of ERK in PLCγ1-driven autophagy of A549 cells. Therefore, AMPKα, mTOR, and ERK signals were involved in PLCγ1 inhibition-driven autophagy in human lung adenocarcinoma A549 cells.

### PLCγ1 inhibition-driven autophagy leads to cell death in human lung adenocarcinoma A549 cells

**Figure 5:**
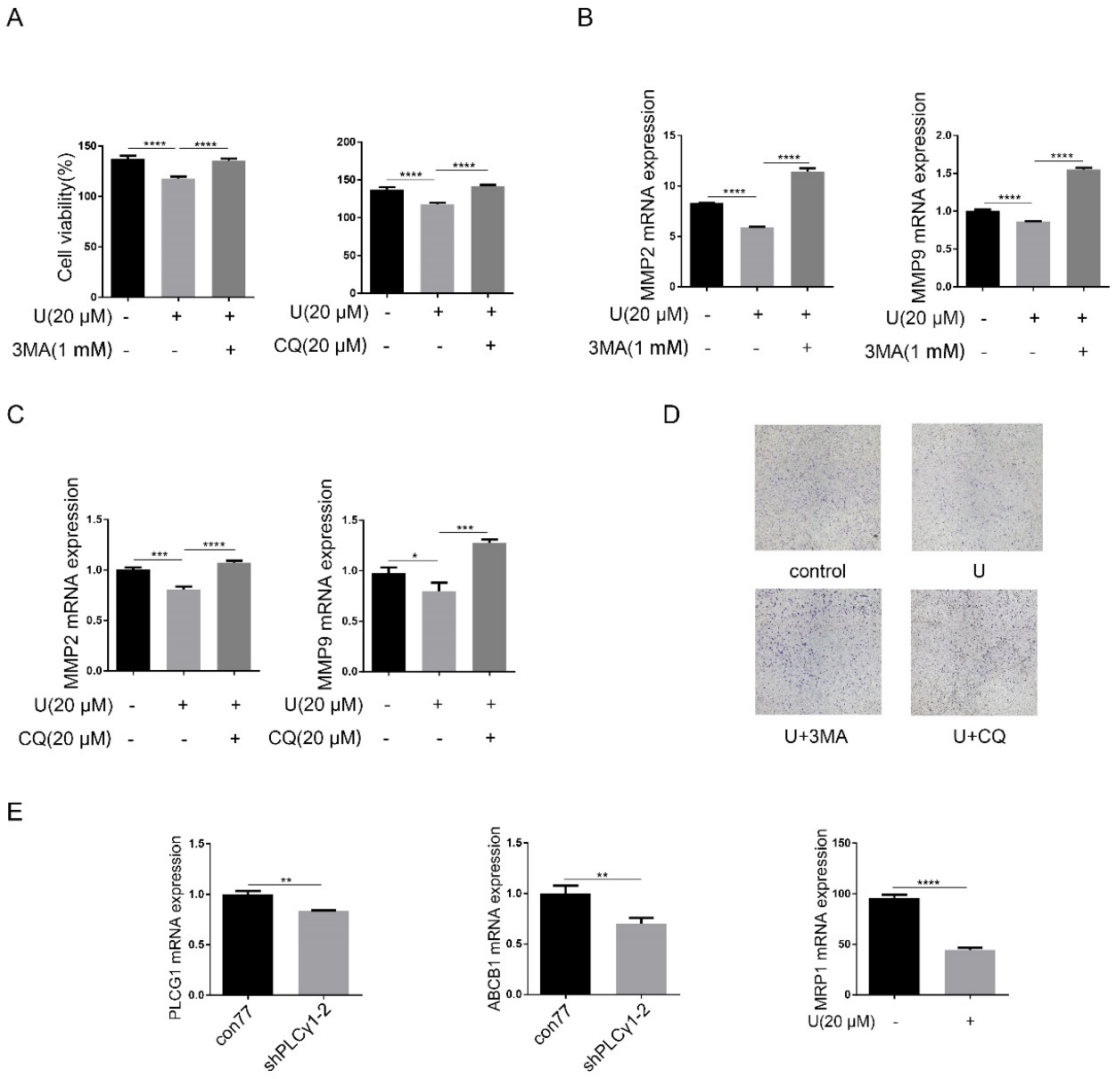
** Effect of PLCγ1 inhibition-driven autophagy on cell proliferation, migration and drug resistance in A549 cells.** (A) & (B) & (C)&(D) Cells were pretreated with or without 3 MA (1 mM) and CQ (20 μM) for 1 hour, respectively, followed by co-treatment with U (20 μM) for 24 hours. The cell viabilities were then detected via MTT assay as described in the Material and methods section (A). The MMP2, MMP9, and GAPDH mRNA levels were detected via RT-PCR assay as described in the Material and methods section (B&C). The migrated cells were observed with Transwell migration assay as described in the Material and methods section (D). (E) Cells were transduced with shRNA/PLCγ1-2 vectors and the PLCG1, ABCB1, MRP1, and GAPDH mRNA levels were detected via RT-PCR assay as described in the Material and methods section. The data are representative of three independent experiments (*p<0.05, **p<0.01, ***p<0.001, ****p<0.0001).

Now that autophagy is a double-edged sword in tumor progression, it is necessary to confirm the effect of PLCγ1 inhibition-driven autophagy on cell proliferation and migration of A549 cells. Fig.5A-D showed that addition of autophagy inhibitors, CQ or 3-MA, reversed the inhibitory effect of U on cell proliferation and migration (***p<0.001, ****p<0.0001, *vs* U-treated group). Considering that increased autophagy in the fully evolved tumors is usually to be thought to contribute to the enhancement of drug resistance, mRNA levels of multidrug resistance-associated protein genes (MRP1) and multidrug resistance phosphoglycoprotein ATP-binding cassette subfamily B(ABCB1) were detected by RT-PCR assay in A549 cells. Fig.5E showed that PLCγ1 inhibition with treatment by U or transduction with shPLCγ1-2 vector led to decreased mRNA levels of MRP1 and ABCB1 in A549 cells, implying that PLCγ1 inhibition-driven autophagy did not enhance drug resistance in A549 cells (**p<0.01, ****p<0.0001, *vs* con77 group).

**Figure 6:**
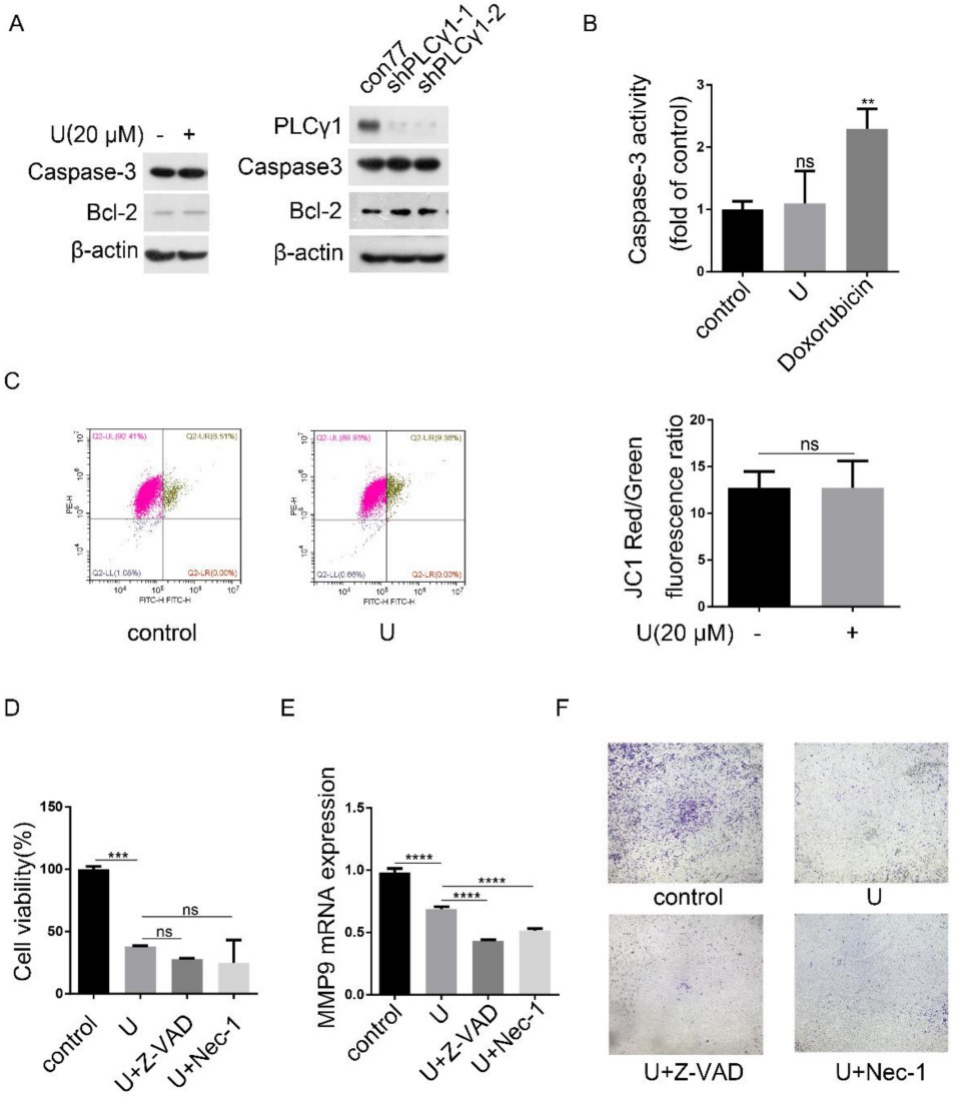
** PLCγ1 inhibition-driven autophagy lead to cell death in A549 cells.** (A) Cells were treated with U (20 μM) for 24 hours or transduced with shRNA/PLCγ1-1/2 vectors, and the PLCγ1, Caspase-3, Bcl-2, and β-actin protein levels were detected via western blotting as described in the Material and methods section. (B) Cells were treated with U (20 μM) for 24 hours (Doxorubicin as positive control). Caspase-3 activity was detected as described in the Material and methods section (B). (C) Cells were treated with U (20 μM) for 24 hours. The mitochondrial transmembrane potential was detected using JC-1 assay as described in the Material and methods section(C). (D)&(E)&(F) Cells were pretreated with or without Z-VAD-FAK (Z-VAD,20 μM) or Necrostatin-1(Nec-1, 50 μM) for 1 hour, followed by co-treatment with U(20μM) for 24 hours. The cell viability was detected with MTT assay as described in the Material and methods section (D). The MMP9 and GAPDH mRNA levels were detected via RT-PCR assay as described in the Material and methods section (E). The migrated cells were detected via Transwell migration assay as described in the Material and methods section (F). The data are representative of three independent experiments (**p<0.01, ***p<0.001, ****p<0.0001).

Programmed cell death includes three forms, autophagy cell death (ACD), apoptosis, and necroptosis, which have respective characteristics 4. Fig.6A showed that either treatment with U or transduction with shPLCγ1-1/2 vectors had not any effect on Caspase-3 expression, but both led to an increase in Bcl-2 level (Fig.6A). Simultaneously, treatment of U did not significantly reduce Caspase-3 activity (Doxorubicin as positive control) (Fig.6B, **p<0.01, *vs* control group). Furthermore, compared with control group, treatment of U did not lead to a decline of mitochondrial membrane potential, implying that the reduction of cell proliferation by PLCγ1 inhibitor might not associated with Caspase-3-dependent apoptosis (Fig.6C). Additionally, neither Caspase inhibitor (Z-VAD-FMK) nor necroptosis inhibitor (Necrostatin-1(Nec-1)) that inhibits allosterically the kinase activity of receptor-interacting protein 1 (RIP1)) treatment influenced the inhibitory effect of PLCγ1 inhibitor on cell proliferation (Fig.6D,* vs* U-treated group).Especially, either Z-VAD-FMK or Nec-1 boosted the effect of U on cell migration in A549 cells (Fig.6E&F, ****p<0.0001, *vs* U-treated group). These results indicated that the reduction of cell proliferation and migration by PLCγ1 inhibition-driven autophagy in A549 cells were different from apoptosis and necroptosis.

### Effect of depletion of PLCγ1 by shRNA or treatment of LiCl on tumor growth and metastasis in a nude mouse xenograft model of A549 cells

**Figure 7:**
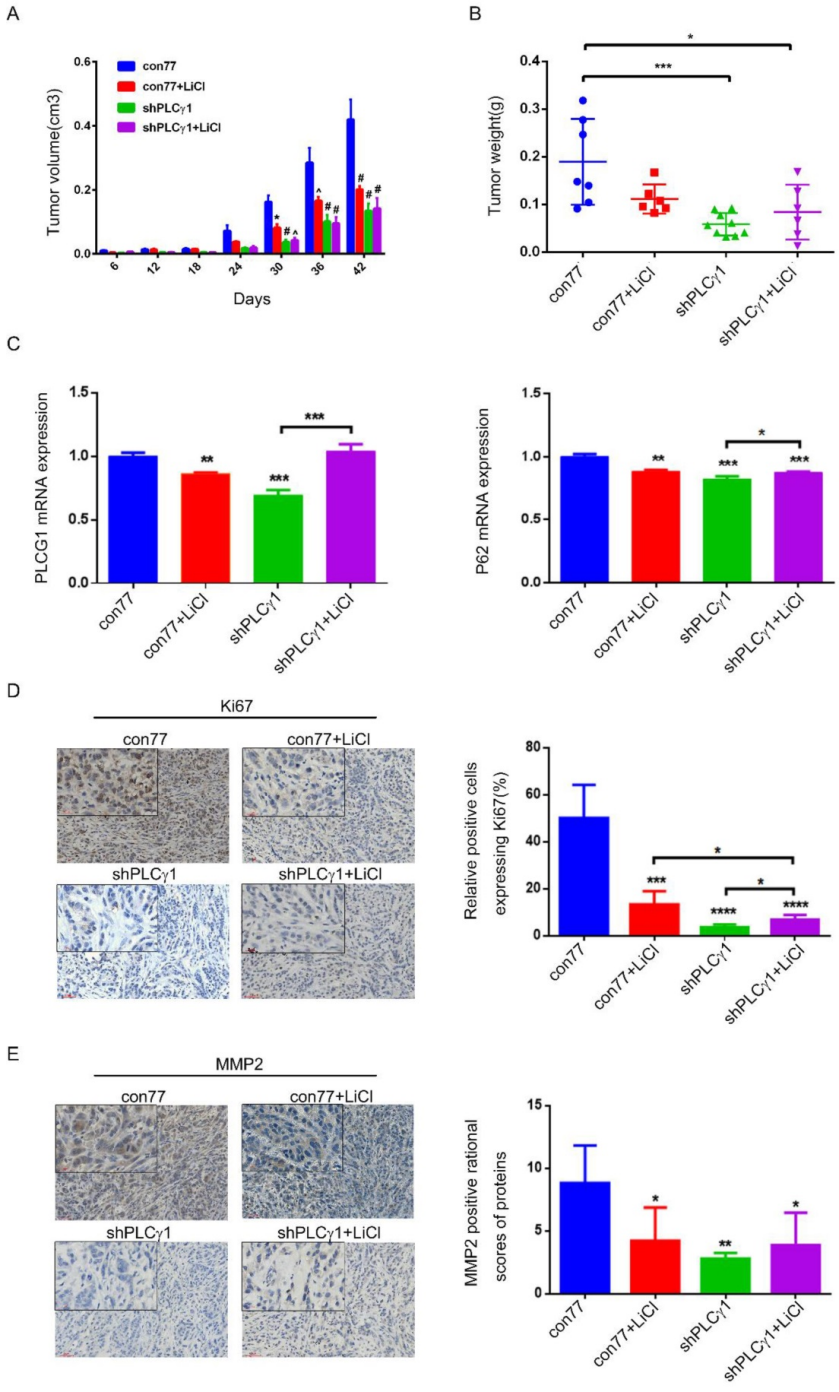
** Effect of depletion of PLCγ1 by shRNA or treatment of LiCl on tumor growth and metastasis in a nude mouse xenograft model of A549 cells.** (A) Volume of tumor sample from nude mice. (B) Weight of tumor sample from nude mice. (C) Relative mRNA level of PLCG1 and P62 in tumor sample was measured using RT-PCR as described in the Material and methods section. (D)&(E) Ki67 and MMP2 protein levels were detected via Immunohistochemistry assay as described in the Material and methods section (*p<0.05, **p<0.01, ***p<0.001, ****p<0.0001).

To determine the role of PLCγ1 and its link to autophagy in the therapy of lung adenocarcinoma, the status of tumor in a nude mouse model harboring tumor xenograft derived from A549 cells transduced with shPLCγ1-2 vector was evaluated. From the beginning of 30 days after subcutaneous injection of A549 cells transduction with or without shPLCγ1-2 vector, subcutaneous tumor volume in con77 group was significantly larger than the other groups (Fig.7A, *p<0.05, ^^^p<0.001, ^#^p<0.0001). Subcutaneous tumor weight was reduced in shPLCγ1 and shPLCγ1+ LiCl groups (Fig.7B, *p<0.05, ***p<0.001, *vs* con77 group). However, there was no significant difference between shPLCγ1 and shPLCγ1+LiCl or con77+LiCl and shPLCγ1+LiCl groups (Fig. 7B). Meanwhile, Fig.7C showed that accompanied with decreased PLCG1 mRNA level (Left panel, **p<0.01, ***p<0.001, *vs* con77 group), mRNA level of P62 in con77+LiCl, shPLCγ1, and shPLCγ1+LiCl groups reduced significantly (Right panel, **p<0.01, ***p<0.001, *vs* con77 group), but shPLCγ1 combined with LiCl led to a significant increase in P62 mRNA level along with increase PLCG1 mRNA level (***p<0.001 in Left panel, *p<0.05, in Right panel,* vs* shPLCγ1 group). The results of immunohistochemistry assay showed that the percentage of relative positive cells expressing Ki67 in subcutaneous tumor tissue of con77 group was higher than that in con77+LiCl, shPLCγ1, and shPLCγ1+LiCl groups (Fig.7D, ***p<0.001, ****p<0.0001). Although the percentage of relative positive cells expressing Ki67 in shPLCγ1+LiCl group was lower than that in con77+LiCl group, the percentage in shPLCγ1+LiCl group was higher than that in shPLCγ1 group (Fig.7D, *p<0.05).Like Ki67 expression in subcutaneous tumor tissue, MMP2 expression level in subcutaneous tumor tissue of con77 group was higher than that in con77+LiCl, shPLCγ1, and shPLCγ1+LiCl groups, while there was no difference between shPLCγ1 and shPLCγ1+LiCl groups or con77+LiCl and shPLCγ1+LiCl groups (Fig.7E, *p<0.05,**p<0.01). Collectively, either the depletion of PLCγ1 by shPLCγ1 or treatment with LiCl could suppress human lung adenocarcinoma growth in a nude mouse xenograft model of A549 cells, but a combination of shPLCγ1 with LiCl did not exhibit more efficacious than one each alone.

## Discussion

Autophagy not only serves as a protective mechanism to facilitate the damaged cellular constituents, but also leads to ACD 21-23. In this study, we demonstrated that inhibition of PLCγ1 promoted autophagic flux and culminated in cell death in A549 cells, in which AMPKα, mTOR, and ERK were involved. PLCγ1 inhibition-driven ACD might contribute to the reduction of cell proliferation and migration by PLCγ1 inhibition in A549 cells.

**Figure 8:**
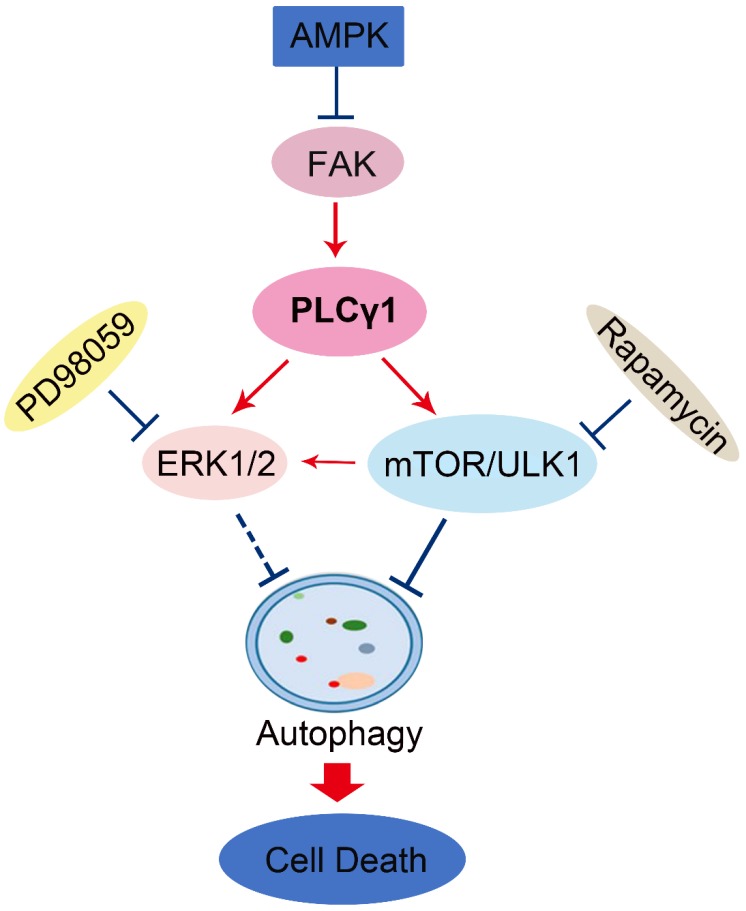
Schematic representation of PLCγ1 inhibition-driven autophagy in human lung adenocarcinoma A549 cells.

Here, our results that addition of CQ interrupted the alteration of LC3B and P62 caused by PLCγ1 inhibition manifested the indispensible role of PLCγ1 in enhancing autophagy flux of A549 cells. At the meantime, the data of integrative and comparative genomic analysis of human lung adenocarcinoma also showed that the negative correlation between PLCγ1 expression and autophagy regulation. Thus, it is conceivable that PLCγ1 inhibition could induce autophagy in lung adenocarcinoma A549 cells as well as in other types of tumors 15, 16. Furthermore, consistent with our previous study, it was observed that PLCγ1 inhibition blocked mTOR and ULK1 phosphorylation in lung adenocarcinoma A549 cells, suggesting thereby that the suppression of PLCγ1/mTOR/ULK1 axis might contribute to PLCγ1 inhibition-driven autophagy. Activated ERK could regulate autophagy either positively or negatively 24-26. Meanwhile, PLCγ1 regulation of ERK phosphorylation has been demonstrated previously 27, 28. Our results that overexpression of PLCγ1 elevated ERK level concomitant with a decrease in LC3B-II and an increase in P62 exhibited the negative regulation of PLCγ1/ERK axis on autophagy in human lung adenocarcinoma A549 cells, thereby suggesting that the inhibition of PLCγ1/ERK axis contributed to autophagy induction in human lung adenocarcinoma A549 cells (Fig.8). In addition, we observed that activated AMPKα with treatment of metformin or transfection with AMPKα vector expressing AMPKα1 and AMPKα2 partially reduced p-FAK and p-PLCγ1 levels in A549 cells, consistent with other authors' and our previous studies 16, 29-32. Therefore, FAK and PLCγ1 might act downstream of AMPK to regulate autophagy in A549 cells (Fig. 8). Most of evidence show that both ERK and mTOR were involved in linear signaling conduits activated by different stimuli and might intersect to regulate each other and co-regulate downstream functions 33. Inhibition of mTORC1 leads to MAPK pathway activation through a PI3K-dependent feedback loop in human cancer 33. mTOR pathway is involved in the post-translational regulation of the ERK phosphatase DUSP6/MKP3 34. IGF-1-stimulated protein synthesis in oligodendrocyte progenitors requires PI3K/mTOR/Akt and MEK/ERK pathway 35. Here, we also found that mTOR inhibitor Rapamycin reduced ERK and p-ERK levels in A549 cells expressing PLCγ1, implying that activated mTOR by PLCγ1 could act upstream of ERK(Fig.8). Consequently, PLCγ1 inhibition could induce autophagy, in which PLCγ1/mTOR/ULK1, PLCγ1/ERK, and AMPK-FAK/ PLCγ1 axes could be involved, as well as the reciprocal regulation of ERK and mTOR (Fig.8).

In general, autophagy enhances cell survival and protects cell against nutrition starvation, DNA damage, and organelle damage 21. However, prolonged or sustained autophagy may induce ACD. For example, oncogenic ras-induced expression of Noxa and Beclin-1 promotes ACD and limits clonogenic survival 22. Isogambogenic acid induces apoptosis-independent ACD in human non-small-cell lung carcinoma cells 23. Here, we observed that autophagy inhibitors reversed the inhibitory effect of PLCγ1 inhibition on cell proliferation and migration, thereby indicating that the inhibitory effect of PLCγ1 inhibition partially depended on PLCγ1 inhibition-driven autophagy. Subsequently, our results that neither Caspase-3 inhibitor (Z-VAD-FMK) nor necroptosis inhibitor (Necrostatin-1(Nec-1) treatment attenuated the inhibitory effect of PLCγ1 inhibitor on cell proliferation and migration indicated that the reduction of cell proliferation and migration caused by PLCγ1 inhibition-driven autophagy was independent on Caspase-3 and RIP1. Therefore, PLCγ1 inhibition-driven autophagy might lead to ACD to reduce cell proliferation and migration in A549 cells, different from usual autophagy occurred in the fully evolved tumors. Recent studies also show that ACD is involved in some tumor therapy. For instance, plant lectins targets both apoptosis and autophagy dependent cell death in cancer therapeutics 36. A novel triazole NMK-T-057 induces ACD in breast cancer cells by inhibiting γ-secretase-mediated activation of Notch-signaling 37. Autophagy induced by ionizing radiation promotes cell death over survival in human colorectal cancer cells 38. Furthermore, addition of autophagy inhibitor, CQ, could sensitize cancer cell to chemotherapeutic drugs 39, 40, then it is possible that simultaneous targeting of PLCγ1 and autophagy pathway might be an efficient new strategy to overcome the autophagy addition of tumor and acquired resistance to current therapy. Disappointedly, our results in a nude mouse xenograft of A549 cells indicated that the depletion of PLCγ1 by shPLCγ1 could suppress human lung adenocarcinoma growth in a nude mouse tumor xenograft model, but a combination of shPLCγ1 with autophagy activator LiCl did not exhibit more efficacious than one each alone. It might be due to the selection of autophagy activator or treatment dose and needs to further study.

In a summary, autophagy induced by PLCγ1 inhibition with PLCγ1 inhibitor or transduction with shRNA/PLCγ1 vectors promoted ACD partially led to the reduction of cell proliferation and migration in A549 cells. PLCγ1 might act not only upstream of mTOR and ERK, but also downstream of AMPK in PLCγ1 inhibition-driven autophagy (Fig.8). A combination between targeting PLCγ1 and autophagy pathway in anti-tumor therapy may be an efficacious new strategy to overcome the autophagy addition of tumor and acquired resistance to current therapy.

## Material and Methods

### Antibodies and reagents

Antibodies against PLCγ1, p-PLCγ1 (Tyr783), Caspase-3, AMPKα, p-AMPKα (Thr172), ERK1/2, p-ERK1/2 (Thr202/Tyr204), mTOR, p-mTOR (Ser2448), p-ULK1 (Ser757), FAK, and p-FAK (Tyr397) were purchased from Cell Signaling Technology Inc. (Beverly, MA, USA). Antibodies against P62, Bcl-2, and ULK1 were purchased from Abcam Inc. (Cambridge, MA, USA). Antibody against LC3B was purchased from Novus Biologicals, Inc. (Littleton, CO, USA). Goat anti-Rabbit IgG and Goat anti-Mouse IgG were purchased from Protein Tech Group (Chicago, IL, USA). The dilution concentrations of anti-Rabbit and anti-Mouse were 1:50000 and others were 1:1000. PLCγ1 inhibitor U73122 (U), autophagy inhibitor PI3K inhibitor 3-Methyladenine (3-MA) and Chloroquine (CQ), MEK inhibitor PD98059, mTOR inhibitor Rapamycin, and AMPKα activator Metformin were purchased from Sigma-Aldrich in China (Shanghai, China). Caspase inhibitor Z-VAD-FMK (Z-VAD) and RIP1 inhibitor Necrostatin-1(Nec-1) were purchased from MedChemExpress USA (Monmouth Junction, NJ, USA). Antibodies against Ki67 and MMP2 were purchased from Ruiying Biological (Suzhou, China) and ABclonal (Wuhan,China) respectively. Other reagents were of the highest grade commercially available.

### Cell culture and treatment

A549 cell and HEK293T cell were purchased from the Shanghai Institute of Cell Biology, Chinese Academy of Sciences (Shanghai, China). Cells were cultured with RPMI 1640 and DMEM high glucose medium (Invitrogen, Carlsbad, CA, USA), respectively, supplemented with 10% fetal bovine serum (FBS), 100 μg/mL streptomycin and 100 U/mL penicillin, at 37°C in a humidified incubator with 5% CO_2_. According to the different experimental requirements, cells were treated by PLCγ1 inhibitor U73122 dissolved in N, N-Dimethylformamide (DMF) and transfected or transduced with plasmids expressing different genes, respectively.

### Plasmid construction and transfection

Short hairpin RNA (shRNA) targeting PLCγ1 (shPLCγ1) was purchased from Gene Chem (Gene Chem, Shanghai, China). ShRNA sequences are shown below, shPLCγ1-1: 5 'CcgggcCATTGACATTCGTGAAATTctcgagATTTCACGAATGTCAATGgcTTTTTg3', shPLCγ1-2: 5'CcggccAGATCAGTAACCCTGAATTctcgagAATTCAGGGTTACTGATCTggTTTTTg3 '. After cells were tranduced with the different shPLCγ1 vectors by a lentiviral transfection strategy, stable cell lines were obtained under the pressure of puromycin (2 μg/mL, BioVision, Inc., Milpitas, CA, USA). In addition, cells were transiently transfected for 24 hours with pRK5-HA-PLCγ1 (pRK5-PLCγ1) vector expressing PLCγ1, p3xFLAG-CMV10-AMPKα1 (AMPKα1) vector expressing AMPKα1, p3xFLAG-CMV10-AMPKα2 (AMPKα2) vector expressing AMPKα2, using Lipofectamine 3000 (Invitrogen, Carlsbad, CA, USA), following the manufacturer's procedure and previous studies 9,41.

### MTT assay

Cells were seeded in 96-well plates and each well contained 100 μL of complete growth medium and cultured for the indicated time. The number of viable cells were measured by 3-(4, 5-Dimethylthiazol-2-y)-2,5-diphenyl-tetrazolium bromide (MTT) assay as described as previous studies 9,42.

### Transwell migration assay

According to the previous studies 9,43, cells in serum-free RPMI-1640 were placed into the top chambers of Transwell inserts set with 8 μm pore filters and RPMI-1640 complete medium was added to the bottom chamber. After 24 hours, cells on top of the membrane were removed with a cotton swab, and then the membrane of Transwell inserts was fixed with 4% paraformaldehyde. The migrated cells on bottom of the membrane were stained with 0.1% Giemsa stain. The migrated cells were observed under an Olympus BX41 microscope equipped with a digital camera (Olympus,Tokyo, Japan) at 4x.

### Colony formation assay (clonogenicity)

As previously described 44, cells were plated in 6-well plates for indicated time. The colonies were fixed with 100% methanol and stained with 0.1% crystal violet, followed with an observation under an Olympus BX41 microscope equipped with a digital camera (Olympus,Tokyo, Japan) at 4x.

### Caspase-3 assay

Cells were seeded in 6-well plates and treated with U (20 μΜ) or doxorubicin (10 μM) for 24 hours. According to the manusfacturer's instruction of Caspase-3 activity assay kit (Beyotime Institute of Biotechnology, Haimen, China), cells were harvested and lyzed in 100ul of the cell lysis buffer included with the kit and protein concentrations were equalized for each condition. Subsequently, 50 μl cell lysate was mixed with an equal amount of substrate reaction buffer containing 10 μl Caspase-3 substrate, acetyl-DEVD-p-nitroaniline (Ac-DEVD-pNA), and incubated for 4 hours at 37°C. Samples were measured by an ELISA reader at an absorbance of 405nm (Infinite F50, TECAN, Swiss) 45.

### Western Blotting analysis

Equal amounts of protein subjected to SDS-PAGE (8-15%) were transferred to a PVDF membrane (GE Healthcare, Hertfordshire, UK) as described in previous studies 46,47. The membrane was incubated with primary antibodies at 4°C overnight, followed by the secondary antibodies at room temperature for 1 hour. An enhanced chemiluminescence (ECL) detection kit (Pierce, Rockford, IL, USA) detected the antibody reactivity.

### Real-time PCR (RT-PCR) assay

Total RNA was extracted using Trizol (Invitrogen, CA, USA) and then subjected to reverse transcription using a Primescript RT Master Mix Kit (Takara, Dalian, China) to synthesize cDNA. Real-time PCR was performed using a LightCycler (Roche) with a SYBR Premix Ex Taq II Kit (Takara, Dalian, China). Results were analyzed as described in previous studies 41,48. The primers used for quantitative PCR are described in Table 2.

### Transmission electron microscopy

Cells were scraped and then pelleted by centrifugation at 2000 × g for 15 min at 4 °C, followed by fixation for 2 h at 4 °C in 2.5% glutaraldehyde in 0.1 M PBS (PH7.4). After scraped and pelleted by centrifugation at 2000 × g for 15 min at 4 °C, cells were fixed for 2 hours at 4 °C in 2.5% glutaraldehyde in 0.1 M PBS (pH7.4) 16,49. Cells were dehydrated and embedded in Embed-812 resin. 70-nm sections were cut using an ultramicrotome (Leica EM UC7, LEICA, Shanghai, China) and then stained with uranyl acetate and lead citrate. Finally, autophagic vacuoles were observed under a transmission electron microscope (Tecnai G2 Spirit BioTWIN, FEI Company, Hillsboro, Oregon, USA).

### Immunohistochemistry assay (IHC)

Samples from subcutaneous tumor tissue and the tissue microarray purchased from Alenabio (Xi 'an, China) for immunohistochemistry assay. As described in the manufacturer's instruction (MAIXIN.BIO, Fuzhou, China), the sections were incubated overnight at 4°C with PLCγ1 (1:100 dilutions), Ki67(1:100 dilutions), and MMP2(1:150 dilutions) primary antibody, respectively. Then the section was incubated with Diaminobenzidine (DAB) and counterstained with hematoxylin. Sections were observed under an Olympus BX41 microscope equipped with a digital camera.

PLCγ1 and Ki67 expressions was evaluated using a semi quantitative scoring system based on staining intensity and the distribution of positive cells. The intensity of protein staining ranged from 0 to 3- i.e., negative (-), weak (+), moderate (++), and strong (+++). Two pathologists were consulted for agreement and the scores were quantified by three independent observers using the criteria for statistical analysis as previously described 50,51.

### Mitochondrial transmembrane potential assay

Briefly, according to the manufacturer's instruction of mitochondrial membrane potential assay kit with JC-1 (Beyotime, China), cells exposed to PLCγ1 inhibitor for 24 hours were treated with JC-1. The fluorescence intensity was measured by flow cytometry (Beckman Cytoflex, USA) with FITC green channel and PE red channel 52.

### Identification of PLCG1 related functions

RNA-seq data of 586 patients diagnosed with lung adenocarcinoma from TCGA dataset were downloaded with the “TCGAbiolinks” R package. Significant related genes with PLCG1 expression were calculated by Spearman correlation analysis in TCGA dataset. Then, the “clusterProfiler” R package was used to analyze the enriched biological process of these genes^57^. In addition, we used GSEA (Gene Set Enrichment Analysis) software to further explore biological functions of PLCG1 in lung adenocarcinoma.

### A549 nude mice xenograft model

Thirty-six 6-week-old female BALB/Cnu/nu nude mice were purchased from Shanghai Slac Laboratory Animal Co. Ltd. (Shanghai, China). All animal studies were conducted according to the regulations of the Institutional Animal Care and Use Committee protocol. This study was approved by the Committee on the Ethics of Animal Experiments of Xiamen University (No.20170196). Animals bearing tumors were randomly assigned to 2 groups (control77 and shPLCγ1-2,18 mice per group). 100 μl relevant stable cells (2×10^6^/mouse) in PBS were subcutaneously injected into the right foreleg of mouse 34, 46. After 6 days post-injection of cells, each group was divided into 2 sections(9 mice per section), one of which was injected with 3 mg/kg body weight LiCl once a day for 42 days and the other was simultaneously injected with the same dose of 0.9% saline. Tumor volume and animal weight were measured every 6 days. All animals were not sacrificed until 42 days post injection. Collected tumor tissue were used for RT-PCR and immunohistochemistry assays.

### Statistical analysis

Differences between the groups were examined for statistical significance using χ^2^ test with SPSS 22.0 software (IHC), Student's *t*-test (between two groups), and one-way ANOVA (among three groups) with GraphPad Prism 6 software. A value of P < 0.05 was considered significant.

## Supplementary Material

Supplementary figures and tables.

## Figures and Tables

**Table 1:** Clinical pathological characteristics of PLCγ1 in human lung adenocarcinoma

	PLCγ1				
	Cases	-/+	++/+++	X^2^	P
**Tumor stage**					
I	28	17	11	8.93	0.003
II, III	28	6	22		
**Metastasis**					
Non-metastasis	33	17	16	3.6	0.057
Regional lymph node metastasis	23	6	17		

**Table 2:** Primers in quantitative PCR

Gene	Forward Primer	Reverse Primer
*PLCG1*	5'-GGAAGACCTCACGGG ACTTTG -3'	5'- GCGTTTTCAGGCGAA ATTCCA -3'
*MMP2*	5'-AGTAAACAGCAAGAGAACCT -3'	5'-ACAGATGCCACAATAAAGC -3'
*MMP9*	5'-ACTACTGTGCCTTTGAGTC -3'	5'- TACTTCCCATCCTTG AACAA -3'
*ABCB1*	5'- CCCATCATTGCA ATAGCAGG -3'	5'- GTTCAA ACT TCTGCTCCTGA -3'
*MRP1*	5'-CCGTGTACTACTCCAACGCTGACAT -3 '	5'-ATGCTGTGCGTGACCAAGATCC -3'
*P62*	5'-TCCCTGTCAAGCAGTATCC-3'	5'-CCTCCTTGGCTTTGTCTC-3'
*GAPDH*	5'-TGCACCACCAACTGCTTAGC -3'	5'-GGCATGGACTGTGGTCATGAG -3'
