# Supplementary material for: Phosphoinositide specific phospholipase Cγ1 inhibition-driven autophagy caused cell death in human lung adenocarcinoma A549 cells in vivo and in vitro
Source: Int J Biol Sci. 2020 Feb 21;16(8):1427–40. doi: 10.7150/ijbs.42962 (PMC7085223; doi:10.7150/ijbs.42962)
Supplement: Supplementary file 1 — Supplementary figures and tables. [file ijbsv16p1427s1.zip › Supplementary Figure legends.docx]

**Figure legends**

**Supplementary Figure S1. IC50 of PLCγ1 inhibitor U73122 in A549 cells.**

**Supplementary Figure S2. Effect of DMF on cell viability in A549 cells**
