# Supplementary figures and images for: Phosphoinositide specific phospholipase Cγ1 inhibition-driven autophagy caused cell death in human lung adenocarcinoma A549 cells in vivo and in vitro
Source: Int J Biol Sci. 2020 Feb 21;16(8):1427–40. doi: 10.7150/ijbs.42962 (PMC7085223; doi:10.7150/ijbs.42962)

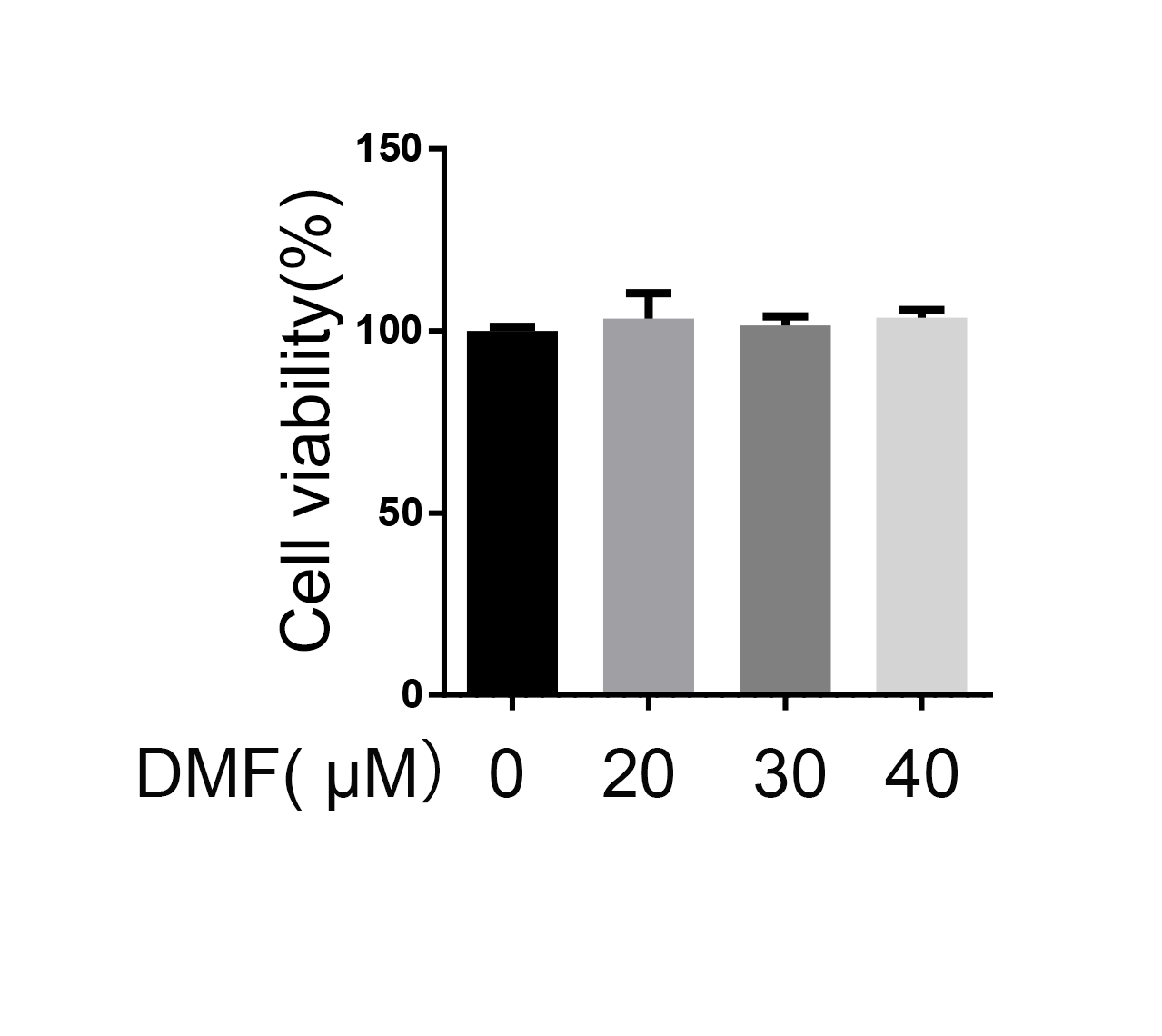

Supplement: Supplementary file 1 — Supplementary figures and tables. [file ijbsv16p1427s1.zip › supplementary FigS2.tif]

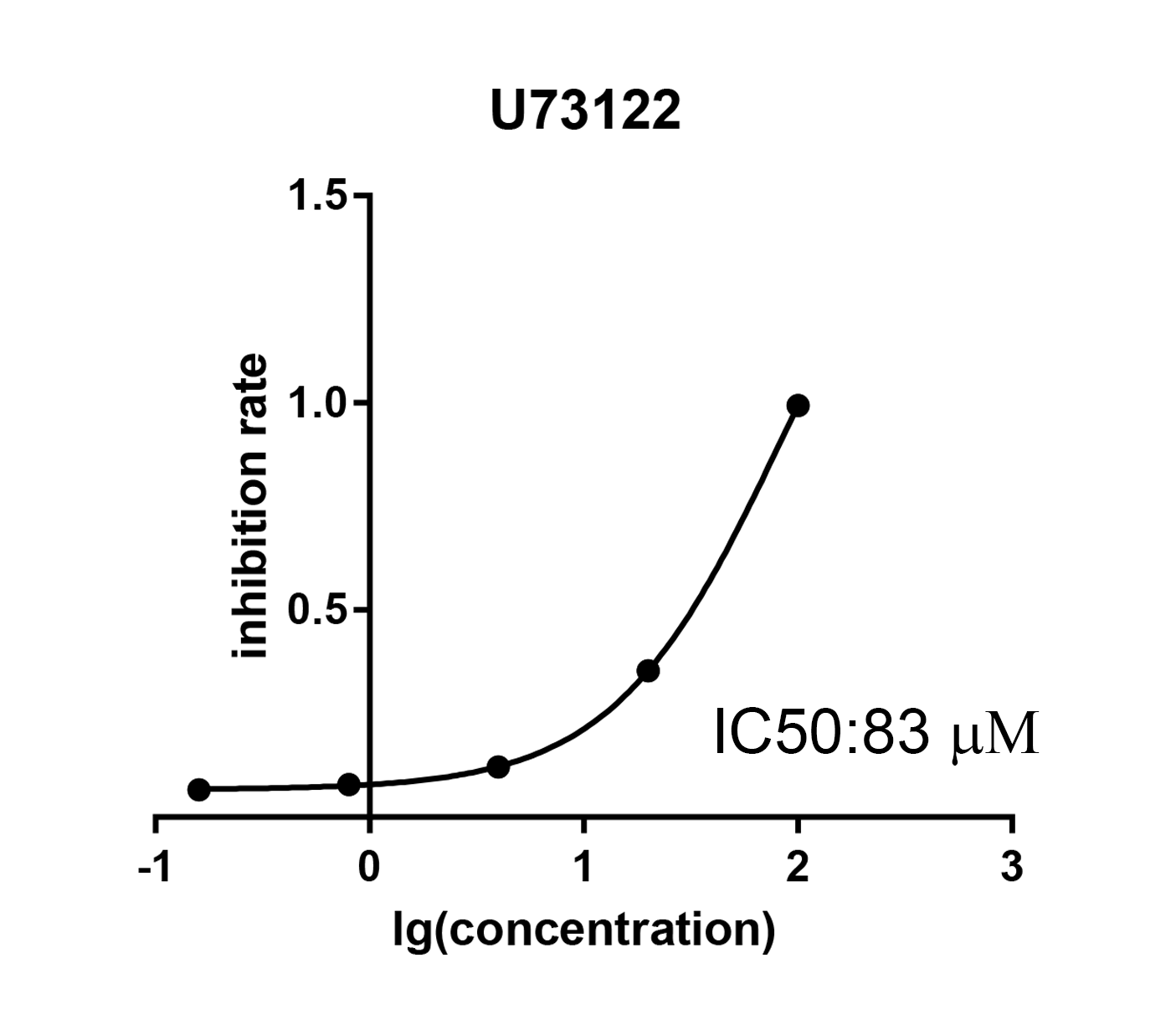

Supplement: Supplementary file 1 — Supplementary figures and tables. [file ijbsv16p1427s1.zip › supplementary FigS1.tif]
